# Reviewing the Composition of Vaginal Microbiota: Inclusion of Nutrition and Probiotic Factors in the Maintenance of Eubiosis

**DOI:** 10.3390/nu12020419

**Published:** 2020-02-06

**Authors:** Antonio Barrientos-Durán, Ana Fuentes-López, Adolfo de Salazar, Julio Plaza-Díaz, Federico García

**Affiliations:** 1 Hospital Clínico Universitario San Cecilio, Servicio de Microbiología, Instituto de Investigación ibs. GRANADA, Avenida de la Ilustración S/N, 18016 Granada, Spain; 2 Department of Biochemistry and Molecular Biology II, School of Pharmacy, University of Granada, 18071 Granada, Spain; 3 Institute of Nutrition and Food Technology “José Mataix”, Biomedical Research Center, University of Granada, Armilla, 18016 Granada, Spain; 4 Instituto de Investigación Biosanitaria ibs GRANADA, Complejo Hospitalario Universitario de Granada, 18014 Granada, Spain

**Keywords:** vaginal microbiome, bacterial communities, vaginal dysbiosis, bacterial vaginosis, risk factors, nutrition, probiotics, hormone replacement therapy

## Abstract

The vaginal microbiota has importance in preserving vaginal health and defending the host against disease. The advent of new molecular techniques and computer science has allowed researchers to discover microbial composition in depth and associate the structure of vaginal microbial communities. There is a consensus that vaginal flora is grouped into a restricted number of communities, although the structure of the community is constantly changing. Certain Community-State Types (CSTs) are more associated with poor reproductive outcomes and sexually transmitted diseases (STDs) meanwhile, CSTs dominated by *Lactobacillus* species—particularly *Lactobacillus crispatus*—are more related to vaginal health. In this work, we have reviewed how modifiable and non-modifiable factors may affect normal vaginal microbiota homeostasis—including sexual behavior, race or ethnicity, and hygiene. Special interest has been given to how the use of probiotics, diet intake, and use of hormone replacement therapies (HRTs) can potentially impact vaginal microbiota composition.

## 1. Introduction

The human body accommodates ecological communities of commensal, symbiotic and pathogenic organisms—known as the microbiota—that reside on surfaces and cavities exposed or not to the exterior environment [1]. The kinds of organisms present include bacteria, archaea, protists, fungi and viruses, and these may differ greatly between body sites and between individuals [2]. The impact that microbiota communities have on the host human body was revealed by studies led by the National Institute of Health in 2008 with the development of the Human Microbiome Project (HMP). Results from this project focused on two main facts: (i) the healthy human body is habited by a large diverse microbiota with more genetic material—a presence that exceeds ours in a 10:1 ratio [3,4]—than the host itself; and (ii) the use of new molecular techniques and statistical methods that use high-performance DNA and RNA sequencing technology instead of culture-dependent techniques make possible the identification of complex microbial communities of microorganisms, demonstrating the great impact of microbiota on the host at different levels: metabolic homeostasis, nutrients acquisition, programmed acquisition of immunity and protection against pathogens among others [1,5,6,7]. In the context of genomics, the term microbiome denotes either the collective genetic material of microbiota microorganisms that reside in an environmental niche or the microorganisms themselves. This term, microbiome, has generated some controversy in the scientific community since its definition. Recently reviewed in [1], it was proposed that this term should refer to an entire habitat that includes the belonging microorganisms, their genomes (i.e., genes), and the surrounding environmental conditions in contrast to the definition that simply considers a mere collection of genes and genomes of the members of a microbiota. The new concept is based on that of “biome”, the biotic and abiotic factors of given environments. It is argued that this is the definition of the metagenome, which, combined with the environment, constitutes the microbiome.

## 2. Materials and Methods

For the review, a search of the scientific literature was conducted using PubMed/Medline with the following search keywords: “vaginal microbiota (or microbiome)”, “vaginal dysbiosis”, “bacterial vaginosis”, “bacterial vaginosis and age”, “bacterial vaginosis and ethnicity (or race)”, “bacterial vaginosis and stress”, “bacterial vaginosis and pelvic inflammatory disease”, “bacterial vaginosis and preterm birth (or pregnancy)”, “probiotics and vaginal microbiota”, and “hormone replacement therapy and vaginal microbiota”. Pertinent original articles and reviews that were peer reviewed, indexed in PubMed/Medline and written in English were included. The publication dates were not limited in order to fully review the literature available until September 2019.

## 3. Results

### 3.1. Human Vaginal Microbiota

#### 3.1.1. Human Vaginal Microbiota: Role as a Natural Barrier

At the histological level, the vagina is a fibromuscular structure that has three main layers or tunics known as mucosa, muscle, and adventitia. The mucous layer forms numerous transverse folds called “wrinkles” or vaginal folds that, in turn, have two layers: stratified squamous epithelium and lamina propria, an unattached connective tissue that joins the epithelium with the muscle layers. Fundamentally, it is in this squamous epithelium where microorganism communities, formerly called the vaginal microbiota, reside. This vaginal microbiota might play a crucial role in gynecologic wellness and in healthy women, consists classically of a diversity of anaerobic and aerobic microorganisms, with the lactobacilli species being the most predominant microorganisms with a determinant function in preventing urogenital diseases such as bacterial vaginosis (BV), yeast infections, STDs, urinary tract infections, and Human Immunodeficiency Virus (HIV) infections [8,9,10,11,12,13,14,15,16,17,18,19,20]. 

The use of new generation molecular sequencing techniques has revealed that vaginal bacterial communities are grouped in three to nine discrete groups—the majority of which are led by lactobacilli [21,22,23]. Ravel et al. [24] analyzed the vaginal microbiota in a cohort of 396 non-pregnant, fertile and asymptomatic North American women from four ethnic groups. Vaginal bacterial communities found in these women were grouped into five main types of CSTs (Table 1). Four of these types of CSTs, found in 73% of women, were dominated by different species of *Lactobacillus* (*L. crispatus*, CST I; *L. gasseri*, CST II; *L. iners*, CST III; and *L. jensenii*, CST V). The last 27% of the communities (CST IV) were varied and formed by a great proportion of obligate anaerobic bacteria, including *Atopobium*, *Gardnerella*, *Prevotella* spp. and other bacterial species (Table 1). These communities are frequently found in asymptomatic healthy women—mainly of the Black and Latin races—but they are also commonly related with high Nugent score [25], a Gram stain commonly conducted in the diagnosis of BV. High Nugent score or changes in the vaginal microbiota have been related with a high risk of STDs, HIV infections, preterm birth (PTB), adverse pregnancy outcomes such as post-abortion sepsis, early, late and recurrent abortions, adverse perinatal outcomes due to PTB and/or histological chorioamnionitis and postpartum endometritis [26,27,28,29,30,31]. Subsequent studies have tuned CTS IV into subgroups IV-A and IV-B (Table 1); both varied in composition, although CST IV-B containing fewer lactobacilli and more anaerobic bacterial taxonomic groups (here *Gardnerella*, *Atopobium*, *Leptotrichia*, *Sneathia* spp. and other organisms related with BV have been included). Many studies have also reported that the important finding that around 20% to 30% of women at any given time have a diverse microbiome deficient in *Lactobacillus*, which historically has not been considered healthy [24,32,33,34]. 

*Lactobacillus* spp. are Gram-positive anaerobic bacteria capable of colonizing the vaginal mucosa, preventing the establishment or excessive development of other microorganisms that may become potentially pathogenic for the host. This protection is performed through two mechanisms: (i) by the specific adhesion to epithelial cells and, (ii) by the production of compounds with antimicrobial properties. In the first place, the ability of the lactobacilli to self-aggregate and adhere to the vaginal epithelium through glycoproteins present on the surface of the epithelial cells (i.e., fibronectin) in a binding that is favored by an acidic pH environment has been described [47]. Although further studies are necessary, it is thought that, in addition to the cellular epithelium of the host, proteins, carbohydrates, glycoproteins, lipoteic acids and divalent cations from microbiota species also play an important role [35].

The presence of lactic acid is key to a healthy homeostasis of the vagina and its production comes from two different sources: by the vaginal epithelium (mainly L-lactate representing 20% of the total lactic acid) and by the microbiota, responsible for metabolizing approximately 80% of glycogen producing the two isoforms of lactic acid with a predominance of D-lactic acid [48,49]. When the squamous epithelium requires energy in the form of ATP, the glycogen from the vaginal epithelial cells is converted to glucose, then to pyruvate, and from this to lactic acid, which is released into the vaginal lumen as the epithelium undergoes desquamation [50,51]. This production of lactic acid is performed under the control of the estrogen levels present in the blood, as these promote maturation and deposition in the vaginal epithelial cells. Therefore, due to the known change in estrogens production throughout the woman’s life cycle, the vaginal ecosystem can be subjected to modifications [50]. The second and main mechanism for producing lactic acid comes from the glycogen found in the vaginal lumen, which is catabolized by alpha amylases to produce maltose, maltotriose and alpha dextrins, which are subsequently converted into lactic acid, due to the action of the Lactobacillus-stimulated lactic dehydrogenase [50,52]. The presence of lactic acid in the vaginal lumen has the consequence that the vaginal pH remains acidic, at levels of approximately 3.5–4.5, generating a protective environment in the mucosa that, partially or totally, inhibits the growth of pathogenic microorganisms [36,53]. Other compounds produced by lactobacilli that play a secondary control in the vaginal flora are hydrogen peroxide (H_2_O_2_) and bacteriocins (reported in Table 1) [36,37]. It has been described that some strains of vaginal lactobacilli can produce H_2_O_2_ protecting the mucosa against alterations caused by opportunistic microorganisms, including those that cause sexually transmitted infections (STIs). On the other hand, bacteriocins are polypeptides synthesized at the ribosomal level whose antimicrobial activity has only been proven in vitro [36,54].

#### 3.1.2. Composition of Vaginal Microbiota Is Defined but Highly Dynamic

Vaginal microbiota composition can be highly dynamic in some women. In short periods, it can go from being dominated by communities led by *Lactobacillus* species to other communities lacking such abundant numbers in these species, while in other women this does not occur, being relatively stable [27]. In both scenarios, there is a certain consensus in the scientific community that vaginal microbial composition has important compositional fluctuations during the woman’s life cycle: birth, puberty, menopause, and transition stages, where steroid sex hormones play a key role in the maintenance of the composition and stability of this microbiota [55,56,57,58]. Among the changes that the vaginal microbiome may undergo, some reports have focused on that these changes may be preferred from one CST towards a specific community condition [34,38]. Also, there is evidence that the CST I community tends to be the most stable in promoting the stability of the vaginal community [22,33,34,38], while CST IV seems to have frequent transitions to many other conditions [38]. On the other hand, it has been reported that a microbiome controlled by *Lactobacillus* species, different from *L. iners* is optimal for vaginal wellness [22,39]. In this sense, it has been shown that the existence of lactobacilli, especially *L. crispatus*, is strongly related with the lack of BV [25,38,39,40]. Very interestingly, it has been observed that the production of lactic acid is an indicative marker in all healthy vaginal communities [59]. Lactic acid has inhibitory properties over pathogenic bacteria [39,56], altering bacterial cell membranes and also improving the host immunity when bacterial lipopolysaccharide is present [60]. In a more precise manner, it has been described that the L-lactic acid isomeric form—either produced by lactobacilli and by epithelial vaginal cells of the host—activates a certain type of immune cells and may encourage that epithelial cells release pro-inflammatory cytokines [61].

Importantly, results obtained on vaginal microbiota composition during pregnancy are of special interest. To date, results are still scarce, and few authors have analyzed the vaginal microbiota composition of pregnant women with methods independent of culture [62,63]. Initially, Verstraelen et al., using a methodology based on Gram stain, culture and terminal restriction of polymorphism fragments, revealed that *L. crispatus* and *L. gasseri* species were important in the maintenance of stable vaginal microbiota in a female population collected once in each trimester [62], being this the agreement accepted and extracted from similar methodologies on the vaginal microbiota during a normal gestation. Based on 16S rRNA gene sequencing methods, several papers are considered of reference. On the one hand, the studies carried out by Romero and collaborators showed that: (i) the vaginal microbiota of healthy pregnant women is different from that of non-pregnant women in composition and stability and, (ii) the microbiota is similar in pregnant women whose pregnancy ended at term or prematurely [64]. Contrary to these latest results, DiGiulio et al. observed variations in vaginal microbiota composition in women who finally had a preterminal termination. Here, an imbalance in the *Lactobacillus* species normally found in the vaginal microbiota was observed, a proliferation of other non-native organisms other than the members of the genus *Lactobacillus*, as well as it was demonstrated that a period of less than 1 year between gestations constituted a high risk of preterm pregnancy due to infection of the amniotic cavity [65].

### 3.2. Vaginal Dysbiosis: An Imbalance in Vaginal Microbiota Composition

Sometimes the concentrations of lactobacilli within the vaginal community are modified, producing an imbalance statement or dysbiosis of the microbiota, which is generally defined as a polymicrobial condition characterized by a low prevalence of *Lactobacillus* spp. and by an increase in anaerobic microorganisms. The most common form of dysbiosis is bacterial vaginosis (BV). This condition is described for three main changes in the environment in vagina [66,67]: (i) a change in vaginal microbiota composition from *Lactobacillus* spp. to facultative anaerobes; (ii) the production of amino compounds by the new bacterial microbiota and; (iii) an increase in vaginal pH to more than 4.5. These are the conditions that mainly favor the development of opportunistic microorganisms that behavior like pathogens, whether they are usually found in the vagina or if they come exogenously [36]. Therefore, diversity in the vaginal microbiota, so-called unhealthy microbiome, is less resistance to alteration and more susceptibility to diseases, including the acquisition of STDs and reproductive and obstetric outcomes [20,33,38,68].

#### 3.2.1. Risk Factors Associated with Vaginal Dysbiosis

**Figure 1:**
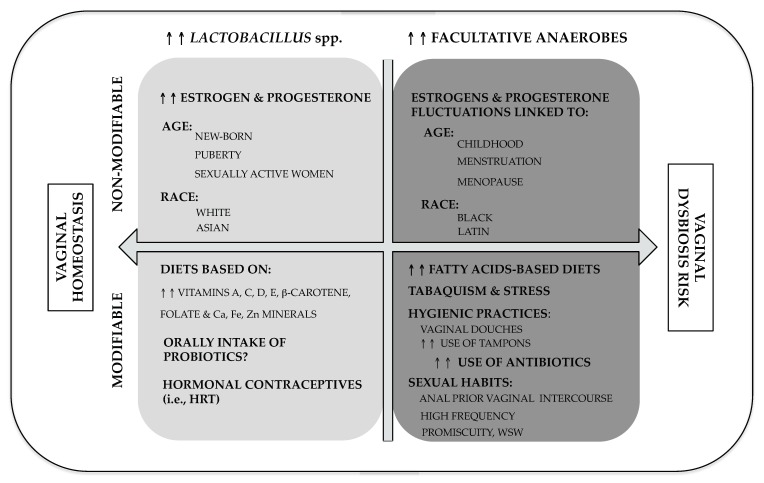
Modifiable and non-modifiable risk factors associated with vaginal homeostasis and dysbiosis. Inherent human conditions linked to vaginal homeostasis and associated with vaginal dysbiosis risk are depicted in the top part of the panel, while modifiable factors are shown in the bottom part. Top and bottom left sections -defined by a double pointed arrow- report those factors that contribute positively to homeostasis. Conversely, right top & bottom sections report those factors associated to vaginal dysbiosis risk. Both of them are associated with a microbiota rich in diverse facultative anaerobes microorganism opposite to those rich in *Lactobacillus* spp. (left sections).

Here, in this section, the main risk factors associated with vaginal dysbiosis (VD) are reviewed. Like in other diseases, risk factors can be categorized as those inherent to the human condition (known as non-modifiable factors) and those related to social conduct or habitats, so-called modifiable factors (Figure 1).

##### Age and Hormone Physiology

Vaginal microbiota composition changes over time. It is well established that vaginal physiology is modified not only due to estrogen production and concentration—which in turn favors the existence of glycogen—but also to vaginal microbiota composition. During pregnancy, it has been thought for years that the fetus develops sterile (now under consideration), and the first microbial colonization occurs at the time of delivery, which comes from the vagina or skin, depending on the route of birth. In newborns, the vulva and the vagina of the infant are influenced by the presence of transplacental estrogenic residues and these favor glycogen supply, which is metabolized by endogenous bacteria, lowering the vaginal pH. As these estrogens are metabolized, a loss of the vaginal glycogen content occurs and thus, the pH is neutralized or alkalized [69]. With respect to childhood, it has been determined that vaginal pH remains neutral or alkaline, with a diphteroid’s colonization (*Corynebacterium* spp. 78%), *Staphylococcus epidermidis* (73%) as well as by M*ycoplasma* spp. [70]. During puberty, maturation of adrenal glands and gonads provoke a rising in the levels of estrogens increasing as well the intracellular production. Two predominant colonies have been determined at this stage of life: *Lactobacillus* spp. and *Atopobium* and *Streptococcus* spp. [70].

In women at reproductive stage, it has been reported that meanwhile menstruation and sexual activity have undesirable consequences on the vaginal microbiota stability—estrogen levels decreased, pH is closed to neutrality which it difficult the growth of lactobacilli (reported in Figure 1)—the secretory phase of the menstrual cycle (described for higher estrogen and progesterone concentrations) is more stable in terms of microbiota composition which correlates high levels of steroid sex hormones [34,71,72]. In the same group of women, hormonal contraceptive administration has been associated with a decrease in the risk of presenting BV, because it generates greater estrogenic stability [36,70,73,74]. Subsequently, as estrogens decrease until menopause, the dominance of *Lactobacillus* decreases and it is stabilized [52]. In postmenopausal women, the decrease in estrogen causes again an increase in pH, which facilitates the presence of enteric bacteria (Figure 1) [75].

##### Ethnicity

It is a fact that the prevalence of suffering from BV, the main consequence of a dysbiotic statement, varies according to the ethnic group. The reasons for the aforementioned differences are not fully understood, although it is speculated that genetic differences determined by the host could govern the composition of species in the vaginal communities [36,52,74,76]. The acquisition of BV has long been associated with black race in the United States (US) (Figure 1) [52,74,75,76] and that this association persists even after using adjustments with variables associated with sexual practices and other confounding factors [52,74,76,77,78]. In other locations such as the United Kingdom and Canada, the prevalence of BV was also higher among Afro-Caribbean and Aboriginal populations, respectively, while in studies performed in countries such as Spain and China, BV prevalence was found higher in Gipsy and Tibetan ethnic groups, respectively [74]. Supporting these results, it has also been reported in other studies about the composition of species in the vaginal microbiome of black and white women born in the US, a significant difference between these two groups, in which black women have a greater microbial diversity and a lower probability of lactobacilli colonization than white women [24,79]. In other studies, conducted in sub-Saharan African countries, a smaller proportion of *L. crispatus* in the vaginal communities compared to women of European or Asian descent has been found [24,79,80]. Here, African communities were dominated by *L. iners* and by a variable mixture of facultative anaerobic bacteria [24,68,81]. Similarly, in a Dutch study about the composition of vaginal microbiome it was significantly associated with ethnic groups where women from African descents had the main occurrence of clusters determined by *Gardnerella vaginalis* or *dysbiosis* [82].

There is even reported evidence that the genetic variation of the host—which can sometimes associate with race or ethnic groups—may be able to affect the microbiome composition. At this point, a large study using metagenomic data from the HMP revealed several associations with key genes of the host related with immune function and abundance of specific microbial taxonomic groups at four distinct locations in the body, although the association with the vagina was not included [83]. Finally, in a retrospective cross-sectional study performed with a small cohort of black South African women, the Black women had a different cervical microbiota without *Lactobacillus* predominance; nevertheless, additional studies are needed to examine whether this microbiota represents abnormal, intermediate or variant states of health [84].

##### Tobacco

Smoking cigarettes has been related with the increased BV prevalence in several epidemiological studies and occasionally in a dose-dependent manner [70,85]. Certainly, a number of compounds resulting from smoking have been identified in the cervical mucus of smokers [70]. Data analysis from sequences have shown an association between smoking and VD even after adjusting for confounding factors (reported within modifiable factors in Figure 1) [79]. In this sense, a 2014 study found two shreds of evidence: (i) that it was significantly more likely that smokers’ vaginal microbiota had a low *Lactobacillus* prevalence and; (ii) metabolites produced during smoking were increased in higher Nugent scores [70]. Recently, the vaginal metabolome of smokers and non-smokers was compared in a cross-sectional study. Smoking was related with differences in vaginal metabolites. Among women categorized to the CST-IV community, biogenic amines were higher in smokers; these amines can affect the virulence of infective pathogens and contribute to vaginal malodor [86].

##### Stress

Stress is defined as any physical or psychological challenge that threatens or has the potential of threatening the balance, homeostasis, of an organism’s internal background [87,88,89]. These challenges can be lifetime events, emotions, and relations that unfavorably affect the individual’s comfort or generate perceived detrimental responses. Very recently, the role of stress over the female lower genital tract has been reviewed [90]. For instance, working with animal models, it has been reported that the persistent exposure to psychosocial stress can lead to an encouragement of the hypothalamic–pituitary–adrenal and sympathetic–adrenal–medullary axes. This, in turn, drives a cortisol-induced inhibition of glycogen deposition in vagina, which is translated into an interruption in epithelial maturation that is crucial to keep vaginal homeostasis (included as negative modifiable factor in Figure 1). This phenomenon is especially relevant during pregnancy, where local production of high levels of corticotropin-releasing hormone occurs in the decidua, fetal membranes and placenta [90].

#### 3.2.2. Other Factors That Influence Vaginal Dysbiosis

##### Sexual Activity

The number of reports published in recent years that try to find associations between human sexual behavior and BV, as the main form of VD, is growing and diverse. Related to the number of vaginal coitus, it has been found that a higher frequency is related with a major risk of suffering from BV [85]. Related to the fact of having multiple, new, or numerous male partners, there is a direct association with BV [81,91]. The maintenance of unprotected sex has been associated with a risk—greater than double—of suffering BV and recurrent BV [91,92], which is adversely related with the quantity and presence of healthy *Lactobacillus* species [81].

Regarding sexual contacts with people of the same gender, a significant relationship between BV and female sexual mates has been found [78], because women in homosexual relationship seem to be at greater risk (Figure 1) compared to women who have heterosexual sex [78,93]. Other reports have studied the impact of certain sexual practices on BV. Although they are moderately limited, strong associations have been found. For example, the association is direct with BV when vaginal intercourse is performed immediately after receptive anal intercourse [85]. On the other hand, there is some controversy about the relationship between receptive oral sex and BV [93]. The increase in the recognition and copy number of *G. vaginalis* genes in the oral cavity among women who have homosexual sex with BV enhances approximately biological plausibleness to a direct association [94]. However, other studies have been unsuccessful to demonstrate such a relationship with receptive oral sex [93]. The controversy persists in studies that seek to find a relationship between BV and receptive oral or anal sex [93]. Finally, digital receptive sex (either vaginal or anal) does not appear to be related with BV [93].

##### Lifestyle and Daily Practices

There are certain types of daily practices that can influence the levels of vaginal acidity, which may significantly predispose the excessive proliferation of opportunistic pathogens [75,95]. These practices can be classified into local and systemic. Within local practices, the use of feminine hygiene products including the use of tampons could alter the vaginal immune barrier having an impact on cellular integrity. Others, like vaginal showers have long been associated with the acquisition of BV (Figure 1). In this regard, longitudinal studies suggest that women who attend these practices have an increased risk of BV incident [96]. The consequences of other intravaginal procedures are not entirely well understood, being some of them more associated with risks of suffering from BV than others [97,98]. Given the heterogeneity of the type of intravaginal practices, the variety of products for this purpose and their wide dissemination among cultures/races, additional research is desirable to clarify the effects on resident microbial communities of vaginal flora [79,81,83]. In addition, it has also been reported that the alkalinity of menstruation or semen neutralizes vaginal pH temporarily and could impact the vaginal microbiota [36,76,90]. Relative to systemic practices, the improper or prolonged use of antibiotics can permeate vaginal exudate, causing alteration in the ecosystem of the vagina. Within this category the aforementioned smoking cigarettes impact and nutrition habits (see below) are also included.

### 3.3. Pathogenesis Associated with Bacterial Vaginosis

As mentioned above, BV is a VD that is defined by a lack of lactic acid—producing lactobacilli and proliferation of facultative and strict anaerobes [99]. BV is the most frequent cause of vaginal discharge [78] and is related with distinct adverse consequences, including an increased risk of PTB, pelvic inflammatory disease (PID), as well as the acquirement of human immunodeficiency virus (HIV) and other sexually transmitted pathogens [20,33,38,68]. Here, in this section, the pathogenesis of BV is reviewed.

#### 3.3.1. Bacterial Vaginosis and Sexually Transmitted Diseases (STDs)

STDs are produced by a wide variety of microorganisms comprising bacteria, protozoa, viruses and fungi. Among bacteria, epidemiological studies have related BV with an increased risk of infection by gonorrhea and chlamydia [20]. In vitro, for instance, it has been proven how vaginal lactobacilli inhibit the growth of *Neisseria gonorrhoeae* [100,101] and other bacterial pathogens [102]. Patients with Nugent scores higher than three were related to a four-fold gain in the gonorrhea infection risk and a triple increase in chlamydial infection risk in a cross-sectional study [15]. In this sense, several longitudinal studies have also established this relationship, being the main study one that shows an augmented risk of chlamydia and gonorrhea incident in women with scores of Nugent greater than 3 [103]. Furthermore, the treatment of asymptomatic BV with intravaginal metronidazole was considerably related with a decrease, by more than triple, in incidental chlamydia in a randomized study [104]; however, recent data from a randomized prospective study showed that detection at home and treatment for BV did not decrease the incidence of chlamydia or gonorrhea [105], results that, taking into account previous research, question the design of this study.

*Trichomonas vaginalis* infection has also been closely associated with BV [103]. In a National Health and Medical Examination Survey performed in 2001–2004, concurrence happened in, around 50% of the women infected with *T. vaginalis* [106]. Trichomoniasis has been linked to low levels of healthy vaginal characterized for the presence of lactobacilli and has been positively associated with an increase in Nugent score [107]. An in vitro evidence shows that the presence of *T. vaginalis* decreases the lactobacilli linked with the epithelium but not the species related with BV [108]. In longitudinal analyses, it has been proven that a Nugent score higher than three was related to a higher risk of *T. vaginalis* infection [109]. To date, few studies use sequencing techniques focusing on the presence of *T. vaginalis* and vaginal microbiome’s composition. In one of these few studies, it was found that the CST-IV community type was considerably related with the detection of *T. vaginalis* [110]. In addition, *T. vaginalis* and BV were independently associated with an increase in the spread of HIV-1 in the vagina, and their concurrence was greatly related with increased probabilities of vaginal spread [111].

BV and herpes simplex virus (HSV) have been epidemiologically connected in multiple cross-sectional and prospective studies. Initial research by Cherpes and colleagues—in a study with 670 women during a year—found that the BV diagnosis was related with a double risk of HSV-2 seroconversion [11]. Subsequently to this, a meta-analysis reported that this relationship could be bidirectional: HSV-2 infection was related in a dependent manner with episodes of BV in sex workers and demonstrated a relative risk of 1.55 for BV incident in women infected with HSV-2 [112]. At the population level, Nugent scores of four or higher have been related with a 32% increase in concurrent HSV-2 and, an 8% increase in HSV-1 [113]. In addition, a meta-analysis has reported that the prevalence of BV was 60% higher among HSV-2 women compared to negative HSV-2 [114]. A recent study revealed that antibiotic-induced VD in mice resulted in a fall of antiviral protection against HSV-2 infection [115]. Furthermore, the association between BV and HSV-2 has also been confirmed in a recent study in South Africa with a large (n = 2750) cohort of patients [116]. In this study, women who had an HSV-2 infection at enrolment were shown to be at increased risk for incident BV infections and, certain risk factors like young age, unmarried and having a partner that has other partners, were significantly related with subsequent BV.

The relationship between BV and Human Papilloma Virus (HPV) is also consistent and well reported in the literature as it is reviewed now. Early longitudinal studies showed a greater relationship of prevalent and incident of HPV in women with both intermediate microbiota and BV [14]. A small, but significant increase in the risk of prevalent HPV, an increase in the chances of incident HPV and late HPV disappearance in women with a Nugent score of seven or higher was reported [117]. In two molecular-based analysis, researchers found that women with HPV positive had a minor fraction of lactobacilli than HPV negative diagnosed women [118,119]. In addition, women with vaginal microbiota dominated by *L. gasseri* appeared to have augmented HPV disappearance rates [119]. Furthermore, other studies have shown that intraepithelial dysplasia severity was significantly related with an increased in microbial diversity in vagina, regardless of HPV condition and showed that the type of community condition had a significant relationship with predominant HPV and that the CST IV-B was linked with HPV positivity [120]. In a retrospective study between 2012 and 2017 with 7081 HPV available cases, authors found that there is a significant association between BV, positive HPV infection, and great-score of squamous intraepithelial lesions [121]. In this study, BV patients with negative HPV infection showed more squamous abnormalities than BV-negative HPV-negative patients [121]. Prevalence of HPV genotypes (HPV59, HPV73, HPV52, and HPV58) increases in women presenting cervical cytological abnormalities has also recently described [122].

There is substantial information that correlates VD with a gained risk of HIV-1 acquisition and transmission. A meta-analysis showed that BV was related with a 60% increment in the risk of contract HIV-1; this comprised four longitudinal studies that inspected HIV-1 incident infection [123]. A model of vaginal mucosa has shown that lactobacilli, predominantly *L. crispatus*, repressed HIV-1 replication [41]. The cervicovaginal mucus with augmented levels of D-lactic acid and a microbiome dominated by *L. crispatus* efficiently stuck HIV-1 in a better way than mucus dominated by distinct microorganisms [41], in addition to the fact that lactic acid at the concentrations obtained in the vagina can incapacitate HIV much effectively in vitro than other acids [124]. Notably, a study in Rwandan sex workers showed that those with a microbiota dominated by *L. crispatus* had a lower incidence of HIV and STIs and that dysbiosis augmented the risk of contracting HIV and STDs in a dose-response manner; in addition, significantly less HIV positive women with microbiota dominated by *Lactobacillus* spp. had demonstrable cervicovaginal levels of HIV-1 [125]. Very recent research has focused on the identification of specific bacterial taxa in the vaginal niche and an increased HIV risk [126]. This analysis demonstrates associations between individual bacterial taxa and pro-inflammatory cytokines (tumor necrosis factor alpha (TNF-α) and interleukin (IL)-1β), suggesting that individual bacterial taxa might show an important role in determining the inflammatory state of the vagina and hence, an increased HIV risk [126].

#### 3.3.2. Bacterial Vaginosis and Pelvic Inflammatory Disease (PID)

PID, infection and inflammation of the uterine lining (endometritis) and fallopian tubes (salpingitis), is a common condition between young women that regularly have the following consequences: tubal factor infertility, chronic pelvic pain and recurrent PID disease [127]. Although PID is a recognized complication of *Chlamydia trachomatis* and *Neisseria gonorrhoeae* infections [128,129], the etiology of up to 70% of cases may be diverse: other cervical, enteric, BV-associated, and respiratory pathogens, including *Mycobacterium tuberculosis* [130,131], may be involved. Truly, early studies revealed that PID frequently occurs in the lack of understood STDs and its etiology [132,133,134]. For instance, in a large longitudinal cohort study it was reported that vaginal transport of organisms associated with BV double increased the risk of incident PID [135]. The application of 16S rRNA bacterial gene sequencing has revealed the presence of specific novel bacterial species in BV [23], some of them are Gram-negative anaerobes such as *Sneathia (Leptotrichia) sanguinegens/amnionii*, have been related in case reports of postpartum fever [42], endometritis [42], tubo ovarian abscesses [136], amnionitis and preterm labor [137] and; Gram-positive anaerobes such as *Atopobium vaginae* has been related with tubo ovarian abscess, tubal factor infertility [43] endometritis [44] and fetal death [45]. Hebb et al. identified bacterial 16S sequences in the fallopian tubes of the 24% of women with salpingitis but in none of the controls [138] including phylotypes closely related to *Leptotrichia* and *A. vaginae*.

In last years, a prospective study has demonstrated that *S. sanguinegens, S. amnionii*, BV-associated bacterium 1 (BVAB1) and *A. vaginae* were related with PID, disappointment of the Centers for Disease Control and Prevention-recommended treatment to eliminate short term endometritis, recurrent PID and infertility, suggesting that optimal antibiotic regimens for PID might need the treatment of new BV-associated microbes [139]. Very recently, in a cross-sectional analysis nested within the PID Evaluation and Clinical Health study has been evaluated if Toll-Like Receptor (TLR) genetic variants are or not related with particular BV-associated microbes that are connected with infertility following pelvic PID. TLRs are part of the native immune system and cooperate in the elimination of pathogens through nuclear factor kappa-b (NF-kB) signaling. Results from this study suggested a modest association of host gene variants in TLR2 signaling pathways with cervical *A. vaginae*—through excessive inflammatory responses—in women with clinical PID [140].

#### 3.3.3. Bacterial Vaginosis and Pregnancy

PTB is the leading cause of neonatal morbidity and mortality and constitutes an important cost cargo on medical management [141]. The PTB etiology is multifactorial although the culmination-shared pathway is always the same. Infection and/or inflammation are major causes [141,142,143] representing up to 40% of the cases [142,144] and probably this is much greater in initial gestations where mortality and morbidity are more frequent [145,146]. BV has been related with adverse repercussions in childbirth. However, the mechanism by which dysbiosis might alter pregnancy continues unclear [147]. Importantly, it has also pointed out that some organisms could affect pregnancy outcomes in a different manner than others and, even that they could impact pregnancy at different gestational ages [148].

Some initial studies have reported that VD cases due to BV in pregnant women have a 5-fold increased risk of PTB before 34 completed gestation weeks [149] and a 7-fold increased risk if BV is detected before 16 weeks [150]. In these cases, clindamycin administration before 22 completed weeks of gestation was related with an 80% reduction in the rate of miscarriage and a significant 40% decrease in PTB [151,152]. Molecular-based techniques have brought a shed of light about how vaginal microbiota impact wellness and illness [153]. The more recent the studies have been conducted, the greater is the association originated between microbiota composition and PTB [38,154,155,156,157,158]. Nevertheless, to date, evidence is limited, and results are, in certain cases, contradictive. Truly, a certain association between preterm labor with diverse vaginal communities has been revealed [38,155]. In this sense, a longitudinal study reported that no woman with delivery at term had CST IV-B [64]. Meanwhile, in a study with a large cohort of pregnant women with intermediate vaginal microbiota, the lack of lactobacilli was related with preterm delivery [159].

In the design of molecular-based studies, the importance of the type of species belonging to *Lactobacillus* genera that resides abundantly on vaginal communities was recently shown. In this study, *L. iners* was significantly overrepresented in vaginal microbiota from women who delivered early preterm (67%), compared to those who delivered late preterm (31%), or at term (29%). On the other hand, *L. crispatus* was related with subsequent term birth compared with early PTB, and a comparatively longer duration of pregnancy than that associated with *L. iners* [160]. Plausible explanation to this association can be because *L. crispatus* takes benefits over *L. iners* with respect to the chirality ratio between the productions of the D- and L-isomer of lactic acid, having these major functional implications [161]. Another hypothesis could be that occasionally *L. iners* might be potentially more pathogenic than a vaginal symbiont [162,163].

### 3.4. Impact of Nutrition in Maintaining Vaginal Homeostasis

#### 3.4.1. Dietary Intake Consequences on Vaginal Homeostasis

Genital tract infections are common in women, with BV being the single most public inferior reproductive tract infection in a population of childbearing age. As already seen here, risk factors for BV include some socio demographic factors, including race and, lifestyle/behavioral factors such as smoking, contraceptive use, douching, sexual behavior and stress. In recent years, researchers have begun to theorize that nutrition is another recognized factor for BV. While little is known about how nutrition may impact vaginal homeostasis, nevertheless, in other body locations such as gut microbiome studies have revealed the surprising effect of the diet on the composition and function of the bacterial community which appears to have a deep impact on human well-being and related diseases including: metabolic disorders, obesity, inflammatory bowel disease and cancer [164]. Furthermore, pro-inflammatory effects of altered intestinal microbiota on distal systems of the body are increasingly recognized [164]. In addition, it is known that the intestine can work as an extravaginal reservoir for lactobacilli and bacteria associated with BV [94].

The investigations published by Neggers et al. and Tohill et al. have constituted the first critical evidence about the role of suboptimal nutrition in BV and other gynecological infections in women of childbearing age. In the first, Neggers et al. have described that subclinical iron and vitamin D deficiencies during pregnancy are related with an increased BV risk [75]. This was also suggested in the studies of Verstraelen et al. [165]. Parallel to the studies, Tohill et al. demonstrated that lesser serum concentrations of vitamins A, C, and E, and β-carotene were associated with BV, and lower iron status was related with increased prevalence of *Candida* colonization in a large cross-sectional study of women with or at risk of HIV. In this work, higher serum zinc concentrations were related with a minor risk of HPV [166].

Subsequently to these studies, Bodnar et al. revealed the contrary relationship between vitamin D and the BV risk during the first trimester of pregnancy [167]. Despite evidence, Klebanoff and Turner [168], in a large longitudinal study, did not find a relationship between vitamin D and BV using a statistical seasonal variable. However, recent results by Akoh et al. again have suggested that minor vitamin D maternal status can increase the infection risk across gestation [169]. Being more precise, authors have observed significant inverse associations between vitamin D and IL-6 and TNF-α in the mother at delivery and between vitamin D and IL-6 and hepcidin in the neonate at birth. Furthermore, authors have revealed that the existence of BV influenced the relationship between IL-6 and vitamin D at delivery suggesting that vitamin D could influence changes in pro inflammatory cytokine production during pregnancy and infections might moderate these relationships. Deeping in the association between nutrients and vaginal health, on certain subsets of women, it has been found an association between the increased of fat in the diet, a higher glycemic load and lower nutritional density [75,170] with BV, and in addition, an contrary relationship between BV and the bigger folate, vitamin E and calcium intake [75]. Besides this, in the latter, the glycemic load was related with the progress and perseverance of BV [170].

BV has also been epidemiologically connected with obesity [78]. In fact, it has been proposed that the increase in saturated fat consumption increases the incidence of BV, and on the other hand, the folate, vitamin E and calcium consumption decreases the BV risk. In the case of pregnant women, iron and vitamin D deficiencies have been related with an increased BV risk [170].

#### 3.4.2. Probiotics Influence on Vaginal Microbiota

It is thought that the vaginal microbiota is mostly formed by the rise of microbes from the rectum. In the vagina, the quantities and categories of residing microbes fluctuate according to certain factors such as hormone levels, sexual contact, douching practices, diet, among others [171]. Vaginal microbiota is a critical actor in gynecologic health, in which bacteria are able to change to a dysbiotic state causing a pathogenic process [172]. BV, the main cause of VD, is the most common genital tract infection in women throughout their reproductive life and it has been related with serious adverse reproductive and obstetric health outcomes, such as PTB and acquisition or transmission of several sexually transmitted agents [170]. Being polymicrobial in nature, BV is considered by a decrease in positive lactobacilli and a significant increase in number of anaerobic bacteria, including *G. vaginalis*, *A. vaginae*, *Mobiluncus* spp., *Bacteroides* spp. and *Prevotella* spp. BV includes the existence of a thick vaginal multi-species biofilm, where *G. vaginalis* is the predominant specie [173]. The standard of-care for BV, an antibiotic therapy based on metronidazole or clindamycin, is incapable to completely eradicate vaginal biofilms, which may explain the existence of high recurrence rates of BV [174]. In addition, prolonged antibiotic therapy can also harm the healthy vaginal microbiota [173]. These issues generated the interesting emerging different therapeutic strategies such as the use of prebiotics and/or probiotics [175]. Probiotics are extensively used to progress gastrointestinal health, but they might also be beneficial to prevent or treat gynecological disorders. In obstetrics and gynecology, probiotics are living microorganisms—mostly formed by *Lactobacillus* spp.—mainly used to restore the physiologic vaginal microbiota in order to treat, besides BV, vulvovaginal candidiasis (VVC) and PTB [176]. Despite this, considerable heterogeneity in probiotic’s effectiveness has been detected during clinical trials [174], which are reviewed in this section.

##### Probiotics in Non-Pregnant Women

The recognized favorable effect of probiotic administration for the BV and VVC treatments has been evaluated in numerous meta-analysis [177,178,179,180] and recently reviewed in [176]. Relative to VVC, it is estimated that approximately seven women out of ten women will live at least one experience of VVC in their lives [181], where recurrence is quite often. This fact has made probiotics a real option to be considered together with current antifungal therapies. In a Cochrane systematic review [180], the efficiency of probiotic treatment for VVC in non-pregnant women was recently under evaluation. The conclusions from 10 randomized controlled trial (RCTs) (1656 participants) studying the influence of probiotics used by oral and vaginal routes, as a coadjuvant therapy to antifungal drugs, were that probiotics slightly enhanced the temporary clinical and mycological cure rate and reduced the 1 month relapse rate. Nevertheless, no influence of probiotic administration was observed on continuing clinical or mycological cure rate (3 month post-treatment evaluation). In addition to this, one of the main and unsolved topics related to the VVC treatment is the extraordinary proportion of reappearances even after the use antifungal (azoles) treatment [182,183], a fact that might be due to augmented presence of azole-drug resistance [184]. For these cases of azole-resistance, it has been proven the effectiveness-protecting role of specific *Lactobacillus* species, an example of this is *L. plantarum* P17630 [184].

Relative to the treatment of BV with probiotics, in 2013, a systematic review by Huang et al. [179] already reinforced the possible favorable effect of probiotics for the treatment of BV. The analysis included 12 RCTs where probiotics were implemented either orally or vaginally with continuation periods from 4 weeks to 6 months. The results revealed that probiotic administration was capable to increase the cure rate in adult BV patients, although some subgroup of analysis failed to prove a positive effect of probiotic administration in long-term treatment (>1 month) [177]. In further analysis, authors investigated the effect of metronidazole administration alone or in combination with probiotics. Five RCTs containing a total of 1186 participants were chosen, and the benefit of combined therapy was proven over metronidazole alone on BV.

##### Probiotics in Pregnant Women

The recognized role of probiotics administered orally on the vaginal niche in the prevention of PTB has been suggested in several studies [185,186]. The rates of PTB differ through different countries, ranging from 5% to 9% in Europe to 13% in US [142]. Although PTB has a multifactorial etiology, it has been expected that approximately one-third of cases are due to intrauterine inflammation [142] triggered by migrant ascending vaginal infections. Remarkably, pre-existing BV give the impression to be intensely related with PTB [187]. Due to this, it has been hypothesized that probiotics could display the possible capacity to transfer and kill resident pathogens in a dysbiotic vagina. Mechanisms in which probiotics might be involved comprise the progress of anti-inflammatory cytokines and the decline of the vaginal pH favoring a vaginal environment that becomes suitable for the growth of healthy bacteria [186,188]. In addition, it has also suggested that, during pregnancy, probiotics might recover maternal glucosidic metabolism over the variation of gut microbial composition and function, as well as an insulin sensitivity improvement [189].

However, the latest published studies do not agree that probiotics have a significantly beneficial role during pregnancy. Some of them are chronologically summarized now. Gille et al. [190] examined the recognized character of oral probiotics on vaginal micro-environment in 320 pregnant women in a triple-blind RCT with oral probiotic supplementation or placebo. After eight weeks of treatment, oral probiotics did not rise the quantity of normal vaginal microbiota compared to placebo.

Subsequently to this work, Jarde et al. [185] have achieved a systematic review and meta-analysis about PTB risk and others unpleasant pregnancy outcomes in pregnant women receiving probiotics. Five studies (1017 women) examined the risk of preterm birth before 34 weeks of gestation, whereas in eleven studies (2484 women) the risk < 37 weeks. Conclusions from these highlighted that the use of probiotics during pregnancy neither decreased nor increased the PTB risk before 34 or before 37 weeks. In addition, it was not seen a protecting effect of probiotic administration over gestational diabetes, preterm premature rupture of membrane (PPROM), and small and large for gestational age infants. Conversely with these results, Daskalakis and Karambelas have previously shown some positive effects in women with PPROM after probiotic administration [191]. In their study, patients were distributed to receive vaginal probiotic in with antibiotic prophylaxis or standard antibiotic treatment alone for 10-days. Women that received the double regimen have higher mean gestational age at birth (35.49 vs. 32.53 weeks) and latency period (5.60 vs. 2.48 weeks) in comparison to control group, although the size sample in this study is questionable (*n* = 59 and *n* = 57, respectively).

Very recently, in a prospective study, Nordqvist et al. [192] evaluated the possible relationship among the probiotic milk consumption and the appearance of PTB and preeclampsia incidences. Maternal inflammatory response is a common background of these two pathologic conditions, and the potential anti-inflammatory effect of probiotics represents the criterion for their selection [193,194]. The study revealed that consumption of probiotic milk in late pregnancy was related with a preeclampsia-reduced risk. Regarding PTB, the probiotic milk ingestion of during early pregnancy was related with a decrease in the PTB risk. In both cases, no dose-response manner was found. Despite these promising results, in both cases, no relationship has been found between the dose applied and the obtained respond. Finally, the results from the studies of Haahr et al. [195] and Olsen et al. [196] do not support the probiotic treatment of BV-positive pregnant women with the objective of (i) diminishing the spontaneous PTB risk and, (ii) reducing the colonization rate of Group B Streptococcal (GBS) on the vagina.

In summary, from the aforementioned latest studies it appears that the use of probiotics during pregnancy neither decreased nor increased the risk of PTB before 34 or before 37 weeks. In a similar manner, no clear profits from the probiotic administration have emerged for PPROM, and for the gestational age of infants.

##### Other Results Obtained with Probiotics

In the success or failure of a probiotic therapy, a good selection of *Lactobacillus* species seems to be crucial. For instance, the putative beneficial effect as probiotic of *L. rhamnosus* BPL005 was recently proven in an in vitro model of bacterial colonization of primary endometrial epithelial cells with the presence of anaerobe microbes such as *A. vaginae*, *G. vaginalis*, *P. acnes*, and *S. agalactiae* [1]. When co-cultured with these pathogens, the *L. rhamnosus* BPL005 was capable at low pH and produced organic acids, producing a significant decrease in *P. acnes* and *S. agalactiae* levels, in contrast, *A. vaginae* and *G. vaginalis* strains were not affected for lactobacilli strain. Furthermore, it has been proven that the *L. rhamnosus* BPL005 colonization in the culture diminished IL-6, IL-8, MCP-1—increased in the existence of pathogens- and raised IL-1RA and IL-1β abundance [172].

### 3.5. Restoration of Vaginal Microbiota through Hormone Replacement Therapy (HRT)

Sex hormones, in particular estrogens, appear to have a significant importance in vaginal health, stimulating the growing of lactobacilli by encouraging glycogen accumulation in the vaginal mucosa [58,197]. In healthy pregnant women, high levels of estrogens contribute to the stability of the microbiota increasing the prevalence of *Lactobacillus* spp. [198]. On the other hand, during menstruation it has been reported a significant microbiota alteration, although this may depend on the type of community [34,40]. Following menopause, the deterioration in estrogen excretion might harmfully affect the vaginal mucosa, leading to vaginal atrophy and reduced glycogen levels that result in low abundance of vaginal lactobacilli. Thus, it has been shown that postmenopausal women who are not under hormonal treatment have significantly inferior free glycogen levels and lower levels and diversity of *Lactobacillus* spp., compared with those using hormonal treatment with higher levels of *Lactobacillus* spp. [199].

In one meta-analysis it was demonstrated that all routes of estrogen administration are effective for relief of menopausal symptoms, especially hot flashes [200]. Focusing in oral administration, one study examined the composition of microbiota of 19 postmenopausal women who were already taking oral estrogen therapy (Premarin-conjugated equine estrogen; CEE). After three months, results from the analysis of vaginal swabs revealed that all the patients were populated by *Lactobacillus* species, especially for *L. iners* and *L. crispatus* [201]. Supporting this, additional studies have found a minor presence of anaerobic bacteria in women under hormonal treatment compared to results from women without a replacement therapy and, equally to first evidence, all women on therapy had *Lactobacillus* existing species in their vagina [202,203]. Focusing in the treatment on symptoms like vaginal dryness and concurrent irritation, a study of women treated with CEE reported improvement subsequently with a treatment of three months, (placebo vs CEE treatment) [204]. Similarly, it has been demonstrated that women who use vaginal estrogen for symptoms of dyspareunia and vulvovaginal atrophy (VVA) score much higher on scales measuring quality of life and sexual health than those women who do not use a hormone replacement-based therapy [205].

### 3.6. Impact of Contraceptives on Vaginal Microbiota

Contraception methods may include the use of estrogen hormones (i.e., estradiol or ethynyl estradiol) or not by progestins, such as medroxyprogesterone acetate (MPA). Routes of administration can be oral, injectable (depot medroxyprogesterone acetace DMPA, or ethinyl estradiol (Net-EN) implants (levonorgestrel or etonogestrel) and intrauterine devices IUDs (such as cupper intrauterine devices).

Relative to BV treatment there is a stable relationship between the use of oral contraceptives and a reduction in BV prevalent [78,206,207]. Together with this latter, a recent meta-analysis has demonstrated a robust undesirable relationship between any hormonal contraception, regardless of type (excluding intrauterine devices), and prevalent, incident, or BV recurrent [208]. However, it has also been reported that certain kinds of hormonal contraceptives may alter vaginal microbiota in a negative manner. For instance, some studies have shown a reduction in prevalent BV in women who use injectable or implanted depot MPA [206]. However, it has also been observed that this contraceptive decreases vaginal Lactobacillus [78,209] and is associated in some studies with an augmented risk of acquisition and transmission of HIV possibly partly intermediated by effects of the microbiota on cervicovaginal inflammation [210]. Comparing the effects on vaginal microbiota from the use of oral contraceptives versus the use of intrauterine systems (IUS) Brooks et al. reported that women using oral contraceptives had a microbiota less colonized by BV-associated microorganisms, meanwhile in patients using levonorgestrel (LNG)-releasing intrauterine systems (IUS) microbiota was colonized by BV-associated microorganisms [211]. Conversely to this latter, Bassis et al. did not find changes in the microbiome consistent with BV in women using the LNG-IUS [212]. Finally, Achilles et al. [213] have recently reported that the use of hormonal contraceptives did not change vaginal microbiota in a period of 6 months, while the use of copper-IUD was related with an increase in the risk of BV and its associated microbiota, including *G. vaginalis* and *A. vaginae* bacteria.

Since contraceptive methods are used extensively by women worldwide, the development of refined research that better elucidates the impact on vaginal microbiota and risk of suffering from BV should be desired. Future research should be focused on precise factors such as the nature of the contraceptives alone or combined with, including a range of applied doses, improvement in routes of administration and extension in the duration of their application—all of them in well-designed controlled population groups of study to achieve more consistent applied results.

## 4. Conclusions

Microbial populations are essential for vaginal wellness. The advance in the characterization of the communities of microorganisms that inhabit the vagina has been extremely fast in recent years although important research gaps still remain unclear. For instance, it is significant to achieve a better understanding of the metabolic interactions between microbiota members and between them and the host. In this regard, multiple studies have begun to clarify the functionality of the microbiome [214] although up to now further evaluation about protein transcription of both microorganisms and the host is needed. This fact will contribute to filling gaps of information over the pathogenesis of interactions between dysbiosis, microorganisms, and the host that lead to adverse clinical consequences, plus to the evaluation of interventions that attempt to maintain or repair a healthy vaginal environment.

The impact of the diet on the composition of vaginal microbiota has also been considered. Being non-inherent in nature, the female population need to start thinking that lactobacilli-based microbiota is favored following healthy practices of alimentation. Summarized here, it has been reported that diets enriched in nutrients such as vitamins (A, C, D, E), B-carotene and minerals (such Ca and Zn) have been positively related with vaginal wellness, including a reduction in the prevalence of BV and HPV. Meanwhile, diets deficient in these nutrients and hence enriched in sugars (glycemic load) or fats (fatty acids) have negative consequences on homeostasis as well as being related with BV [75,165,166,167,168,169,170].

BV is the most frequent single infection of the lower reproductive tract. Since BV current cure rates range between 50% and 80% after treatment with metronidazole, recurrence being very common [215], more effective treatments are needed. The consequences of the alteration of the biological films—mainly colonized by anaerobic *G. vaginalis*—[216] and the benefits of the administration of probiotics [217] should be studied in more detail to achieve a better cure and prevention of recurrent infections, respectively. The primary aim of probiotics in obstetrics and gynecology is the restoration of a functional vaginal microbiome. However, given the inconclusive results for the use of probiotics, some international guidelines, such as the Centers for Disease Control and Prevention [218], do not support the use of any available lactobacilli-based formulations as probiotics as coadjuvant therapy in women with VVC and BV. Very surprisingly, the guideline for probiotics differs between countries, without a universal background [219]. Indeed, if probiotics are prescribed in the treatment with specific disorders, they should be regulated as drugs rather than foods or supplements. Under this formula, adverse consequences connected to the use of probiotics should be shared and registered by health authorities [220]. Nowadays, probiotic effects seem to be strain specific and dose dependent, and the lack of standardized manufacturing procedures affect multiple factors such as microbial survival, their growth, and their viability [220]. At the research level, active work in the field is needed and well-designed studies in the future should also focus on other aspects such as: (i) the efficacy and search of distinct mixtures of strains of probiotic species in the restoration of vaginal microbiota, (ii) a consensus in the duration of the treatment with probiotics and colony-forming units employed for restoration in launched studies, and (iii) a better understanding of the combination of antibiotics and probiotics when both are provided together [39].

Relative to risk factors associated with BV and other pathogenesis linked with dysbiosis, from now and in the immediate future, the performance of studies that focus on the impact of social sexual networks in the conformation and transmission of the vaginal microbiota and the prevalence of BV is significant. Given the importance of the structure of current social sexual networks for the transmission and prevalence of STDs [221], it is possible that these factors are similarly significant in the composition of the vaginal microbiota. For instance, it would be necessary to conduct further comprehensive longitudinal studies based in the consequences and effect of overlapping couples and how the duration of concurrent couples may have on the vaginal microbiota in distinct populations and cultures [46,74]. These studies could greatly contribute to explaining that racial differences are seen consistently in vaginal microbiota. In addition, other studies that focus on sexual habits, such as order of sexual acts and coital frequency might contribute to explaining variances in the composition of the vaginal microbiota and, in parallel, might facilitate relevant information to reduce risks of dysbiosis for women. The treatment of sexual companions of women with recurrent BV has not diminished recurrence in several RCTs, although this could be due to limitations of the study design and ineffective treatments [221] so profound research would be needed on the efficiency of the management of sexual partners. In addition, the mode of birth effect on the creation and maintenance of a healthy vaginal microbiome may be important an important research area since it has been shown that cesarean sections significantly affect the composition of the intestinal microbiome [222,223], and thus its possible influence on vaginal health.

Hormone replacement therapy-based studies outlined herein reported women having a vaginal microbiota dominated by *Lactobacillus* species, which corroborates that levels of estrogens have a profound effect on vaginal community and structural bacteria. Indeed, estrogens not only improve vaginal symptoms such as dryness and VVA but permit re-colonization of the postmenopausal vagina with lactobacilli and, hence, reduce the risk of BV and VVC among others. Hormone replacement therapy has also been correlated to improve sexual quality of life of postmenopausal women, perhaps linked to the aforementioned lactobacilli presence; however, there is a lack in holistic studies that correlate fluctuations in the vaginal microbiota directly to improved sexual wellness and quality of life [224]. Special care with the hormone replacement therapy (HRT) should be taken, cause not all the formulations works properly, as seen here in the case of medroxyprogesterone acetate [206] treatment with negative consequences for vaginal microbiota.

Contraception is a widely used practice in women worldwide and thus knowing how it impacts on microbiota is of great importance. To date, hormonal contraception seems to have more beneficial results over vaginal homeostasis and hence diminishing the risk of suffering from BV, by mean of favoring a lactobacilli-based microbiota as reported in [211]. Conversely, research about the use of IUs (i.e., levonorgestrel (LNG)-releasing intrauterine systems or Cooper intrauterine device, Cu-IUD) has revealed that even at mid-term (i.e., 180 days) abundance of anaerobic bacteria associated with BV increase and scoring higher in Nugent Gram stain [212,213]. However, frequently found in the literature are weak points of research related to the presence of contraceptives (if alone or combined with), the dose applied, questioning the routes of administration and the difficulties for the selection of controlled groups of population to perform very consistent results.

## Figures and Tables

**Table 1:** Organization of the vaginal flora into community types (CSTs) in asymptomatic non-pregnant, sexually active women and their contribution to homeostasis (wellness) or vaginal dysbiosis (disease).

CST	Vaginal pH	Ethnic Group	Type of Bacteria	Microorganism’s Contribution to Homeostasis or Dysbiosis	References
**I**	4.0 ± 0.3	White	*L. crispatus*	*^1^Lactobacillus* spp. beneficial impact reside on: Preventing urogenital diseases. Adhesion to epithelial cells. Production compounds with antimicrobial properties (i.e., H_2_O_2_) Stimulated Lactic Dehydrogenase→Decrease pH→Protective Environment. Production of Bacteriocins. ^2^Common properties linked to dysbiotic statement:	[8,9,10,11,12,13,14,15,16,17,18,19,20,22,25,35,36,37,38,39,40,41]
**II**	5.0		*L. gasseri*		
**III**	4.4	Asian	*L. iners*		
**IV A**			*G. vaginalis* *A. vaginae* *Prevotella spp.*	Production of Biofilms → Adhesion to Epithelial Cells→ Antibiotic Tolerance, Resistance to Host Immune Defence. Production of Cytolysins. Production of Amines → pH Alkalinisation. Activates NF-κB cascade. Secretion of Collagenase and Fibrinolysins→Enhance Mucosal Surface Degradation →Detachment of Epithelial Cells.	[42,43,44,45,46]
	5.3 ± 0.6	Black Hispanic		^3^ Common properties linked to dysbiotic statement:	
**IV B**			*A. vaginae* *Leptotrichia spp.* *Mobiluncus spp.*	Secretion of Collagenase and Fibrinolysins→Enhance Mucosal Surface Degradation →Detachment of Epithelial Cells. Adhesins that contribute to Epithelial Colonization. Hemolysin→Cytotoxic Activity. ^3^ plus: Malic Acid and Trimethylamine Production→vaginal irritation.	[42,43,44,45,46] [46]
**V**	4.4		*L. jensenii*	^1^	[8,9,10,11,12,13,14,15,16,17,18,19,20,22,25,35,36,37,38,39,40,41]

From left to right, columns depict: organization into CSTs, associated pH, Human Races attributable to, predominant microorganisms of these CSTs, properties of them that impact to homeostatic or dysbiotic statement and, given references from literature. Five CSTs are generally accepted, being four of them dominated by different species of *Lactobacillus* (*L. crispatus, L. gasseri, L. inners and L. jensenii*), which are associated to a healthy statement of the vagina (referred as homeostasis). CST IV is split into CST IV-A and IV-B communities where *Lactobacillus* spp. are not predominant and by contrast, a diversity of facultative anaerobes have been identified. Main members of these subgroups are species of: *Gardnerella, Atopobium, Mobiluncus, Prevotella and Leptotrichia.* Diversity of these two sub-CSTs is linked to a dysbiotic statement being BV the most single common manifestation of disease (mentioned in the body text). Common properties of *Lactobacillus* spp. contributing to vaginal wellness as well as features of anaerobe microorganisms that contribute to vaginal colonization and, hence, to dysbiosis are given. This table is an updated and adapted version of that published by Ravel J et al. [24].
